# Revisiting the impact of foreign portfolio investment on stock market performance during COVID-19 pandemic uncertainty: Evidence from India

**DOI:** 10.1016/j.mex.2022.101988

**Published:** 2022-12-29

**Authors:** K.P. Prabheesh, Sanjiv Kumar, Ameen Omar Shareef

**Affiliations:** Dept. of Liberal Arts, Indian Institute of Technology, Hyderabad, Kandi, Sangareddy, Telangana 502285, India

**Keywords:** Stock returns, Foreign portfolio investment, COVID-19-uncertainty

## Abstract

This paper re-examines the causality between stock returns and foreign portfolio investment (FPI) flows in the Indian context during the COVID-19 pandemic. Using the Covid-19 index constructed by Narayan et al. [19] and the Toda and Yamamoto Granger causality test, the study reveals that bi-directional causality runs from FPI flows to stock returns in the early period of the Covid-19 pandemic. Whereas after the peak of the pandemic, there is a unidirectional causality that runs from FPI flows to stock returns.

• Bi-directional causality runs from FPI flows to stock return during the initial period of COVID.
• In the second period, unidirectional causality runs from FPI flows to stock returns

## Graphical abstract

**Figure:**
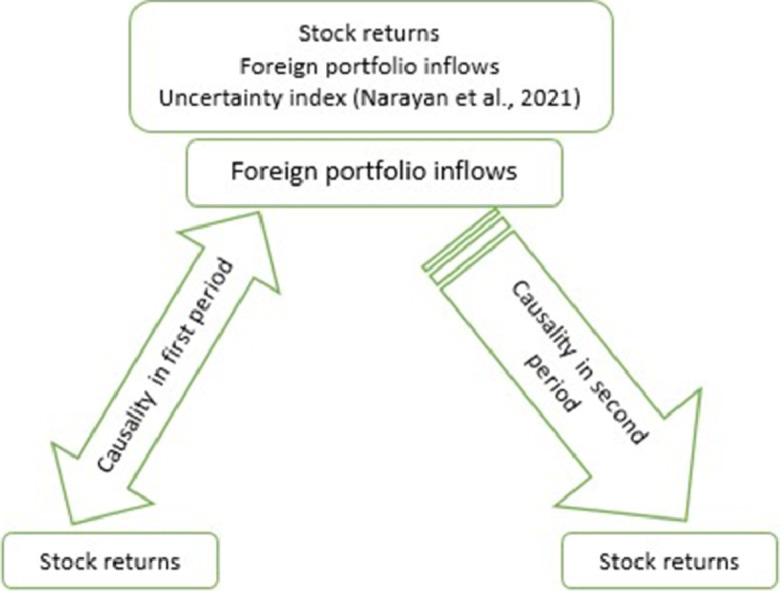

Specifications Table

**Table:** 

Subject area:	Economics and Finance
More specific subject area:	Stock markets, foreign portfolio inflows, uncertainty
Method Name:	New Measure of the COVID-19 Pandemic: A New Time-series Dataset
Name and reference of Original methods:	Narayan, P. K., Iyke, B. N., & Sharma, S. S. (2021). New measures of the COVID-19 pandemic: A new time-series dataset. *Asian Economics Letters, 2*(2), 23491. https://a-e-l.scholasticahq.com/article/23491-new-measures-of-the-covid-19-pandemic-a-new-time-series-dataset
Resource availability:	N.A.

## Method details

In order to check for the granger causality, literature has mainly relied on vector autoregression (VAR) and error correction model (ECM) methods. If the selected time series variables do not possess co-integration properties, then we apply the VAR model. If the variables are known to be co-integrated, then the ECM model is utilized. It implies that we need to ensure the order of the integration of selected variables by doing an initial test on whether they are integrated, co-integrated or stationary. Toda [34] argued that the pre-tests for co-integration in Johansen types of ECM model are very sensitive to values of nuisance parameters in a fixed sample. Therefore, causality interpretation might be influenced by pre-test biasness [38]. To overcome the problem, Toda and Yamamoto [34] have proposed the augmented VAR types model to test for causality, which can be applied to any arbitrary level of the order of integration. Zapata and Rambaldi [37] argued that when there is uncertainty about whether the selected variables are I(0) or I(1), then the preferable model is to apply the Toda and Yamamato causality test because it has higher power in moderate to large sample sizes.

Given its unique characteristic, such as high power, more suitable to moderate to large sample sizes and does not need to worry about the order of the variables; thus, it is widely applied in the literature. Hence, this study employs the Toda and Yamamoto granger causality test to analyze the causality between the Impact of Foreign Portfolio Investment on Stock Market Performance during the COVID-19 Pandemic Uncertainty. This procedure in equation form can be represented in the following form: Let us assume that $Y_{t}$ be k vector variables, which can be obtained through the following model:

(1) $$z_{t}=\gamma_{0}+\gamma_{t}t+\varepsilon_{t}$$

With the term $\varepsilon_{t}$ is following VAR(q) process can be written in equation form as follows:

(2) $$\varepsilon_{t}=\delta_{1}\varepsilon_{t-1}+\ldots +\delta_{q}\varepsilon_{t-q}+\mu_{t}$$

Where $\varepsilon_{t}$ is a white noise error term. By substituting $\varepsilon_{t}=z_{t}-\left(\gamma_{0}+\gamma_{t}t\right)$ from Eq. (2) into Eq. (1), we get the following term.

(3) $$z_{t}=\zeta_{0}+\zeta_{1}t+\delta_{1}z_{t-1}+\ldots +\delta_{q}Z_{t-q}+\mu_{t}$$

$\zeta_{0}$ are function of $\gamma_{i}$ and $\delta_{j}$ with i=01, and j=1,2,3,…q.

The lag-supplemented VAR model advocated by Toda and Yamamoto [34] and Dolando and Lutkepohl [5] to estimate the Granger causality test for a possible integrated variable $z_{t}$ can be written as follows:

(4) $$Z=\tau \Gamma^{\prime }+P\Theta^{\prime }+R\phi^{\prime }+\varepsilon$$

Where $Z={\left(z_{1},\ldots ,z_{t}\right)}_{T\times n^{\prime }},$ $\tau ={\left(\tau_{1},\ldots ,\tau_{t}\right)}_{T\times 2^{\prime }}$ $\tau_{t}={\left(1,t\right)}_{2\times 1^{\prime }}$, $P={\left(P_{1},\ldots ,P_{T}\right)}_{T\times nq^{\prime }}$, $p_{t}={\left(z_{t-1}^{\prime },\ldots ,z_{t-q}^{\prime }\right)}_{nq\times 1^{\prime }}$, $\Theta ={\left(\delta_{1},\ldots ,\delta_{p}\right)}_{n\times nq},$ $R={\left(r_{1},\ldots ,r_{T)}\right)}_{T\times nd^{\prime },}$ $r_{t}={\left(z_{t-q-1^{\prime },\ldots ,}z_{t-q-d^{\prime }}\right)}_{nd\times 1^{\prime }}$, $\Phi ={\left(\delta_{q+1},\ldots ,\delta_{q+d}\right)}_{n+nd}$ and $\varepsilon ={\left(\varepsilon_{1},\ldots ,\varepsilon_{T}\right)}_{T\times n^{\prime }}$where d is the highest order of interation of $z_{t}$.

The wald of the restriction imposed by the null hypothesis

$$H_{0}:S\psi =0$$

(5) $$W={\left[S\hat{\psi }\right]}^{\prime }{\left[S\left(\hat{\Omega }⊗{\left(P^{\prime }QP\right)}^{-1}\right)S^{\prime }\right]}^{-1}\left[S\hat{\psi }\right]$$

Where $\hat{\psi }=vec\left(\hat{\Theta }\right)$ represents the row vectorization with $\hat{\Theta }$ is the OLS estimator can be estimated by $\hat{\Theta }=P^{\prime }QP{\left(P^{\prime }QP\right)}^{-1},Q=Q_{t}-QR{\left(R^{\prime }Q_{t}R\right)}^{-1}R^{\prime }Q_{t}$, where $Q_{t}=I_{t}-\Gamma {\left(\Gamma \Gamma^{\prime }\right)}^{-1}\Gamma^{\prime }$ with $I_{T}$ is the identity matrix. The term $\hat{\Omega }=T^{-1}{\hat{\varepsilon }}^{\prime }\hat{\varepsilon }$ and S is a $m\times n^{2}q$ matrix, where m is restrictions. The Wald statistic is asymptotically $\chi_{m}^{2}$ under the null hypothesis and with conditional homoscedasticity assumption [11].

The steps to estimate the causality through Toda and Yamamato are as follows:

(1) Find the maximum order of integration (d) of selected variables using conventional unit root tests.
(2) Identify the optimal lag length q of a VAR model, which can be estimated using the Akaike information criterion (AIC) and Bayesian information criterion (BIC) lag length criterion.
(3) Estimate the lag-supplemented VAR (*q* + d*)* model:
  $$z_{t}=\zeta_{0}+\delta_{1}z_{t-1}+\ldots +\delta_{q}Z_{t-q}+\mu_{t}$$
  where ζ is a vector of constant, $\delta_{t}$ is the coefficient matrix, and $\mu_{t}$ represent the white noise error term.
(4) Check for the robustness of VAR (*q* + d*)* by employing various available diagnostic measures.
(5) Finally, a Wald test is conducted on initial q parameters, and that provides an asymptotic Chi-square distribution with q degrees of freedom (for more information, follow [34], and [38]).

## Introduction

**Fig. 1:**
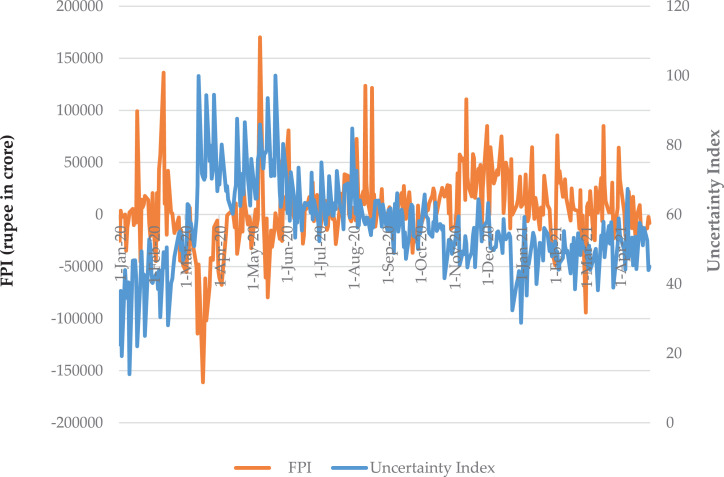
Trends in Foreign Portfolio Investment in India and Covid-19 Index.

**Fig. 2:**
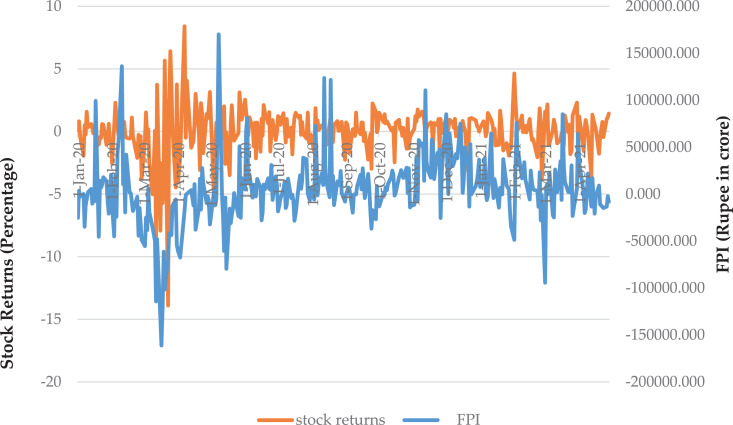
Movements in stock price returns and Foreign Portfolio flows in India.

Emerging market economies (EMEs) financial markets are often sensitive to global financial conditions. Thus, international capital flows play a significant role in determining the macroeconomic conditions of these economies [22]. As EMEs’ stock markets are well integrated with the global capital markets, the sudden inflow and outflow of foreign portfolio investments create significant volatility in their stock markets [20]. It is also argued that EMEs’ asset prices are more sensitive to international market environments than domestic policy changes [30]. The outbreak of the COVID-19 pandemic in early 2020 created uncertainty in the global markets; thus, most of the EMEs stock markets experienced a drastic downfall due to the reversal of portfolio flows worth more than $100 billion within a month of the pandemic [12]. Fig. 1 shows the co-movements of the COVID-19 uncertainty index proposed and constructed by Narayan et al. [19] along with FPI to India. It can be observed that a strong negative co-movement between the two, especially from Jan 2020 to May 2020, indicates that COVID-19-induced uncertainty adversely affected the foreign capital flows to the Indian economy. However, as the covid-19 index declined after May 2020, the portfolio flows were found to be more stable. Similarly, Fig.2 shows the trends in stock returns and FPI, further indicating the Indian equity market co-moves with the FPI flows. Both these figures suggest that the COVID-19 outbreak increased the dynamics of FPI and stock market performance in India. Thus, this paper analyses the dynamic relationship between stock returns and FPI flows in the presence of COVID-19 uncertainty.

Opening up the capital markets to foreign investors carries pros and cons. The key advantages of attracting foreign investment would reduce the cost of capital [7] and risk distribution among domestic and foreign investors [2,9]. Similarly, it also helps to reduce the gap between savings and investment and provides needed foreign exchange to finance current account deficit [8]. Likewise, foreign capital also helps to improve the valuation of the markets as the foreign investors possess superior information [1] and improve the market efficiency (Todea and Pleşoian, 2013). In contrast, attracting foreign capital flows to the financial markets can also lead to higher volatility in the financial markets as these flows are susceptible to rapid reversals and abrupt stops [20]. Further, foreign investors follow approaches that make stock prices overreact to changes in the macroeconomic fundamentals [6]. The trading strategies, such as positive feedback trading, i.e., the foreign investors who purchase the shares as prices surge and sell as prices decline, can destabilize the stock market [3,6]. Similarly, during the financial crisis, the sudden withdrawal destabilizes the stock market due to the short-term speculative behavior of foreign investors [16,33].

Numerous studies show the adverse effect of COVID-19 on stock market movements ([4,^10^,[13], [14], [15],^17^,^22^,^23^,29]; and [21,[24], [25], [26], [27], [28],^31^,^32^,^35^,36]). However, the impact of the behavior of foreign investors in the course of the COVID-19 pandemic on stock market movements is rare. One study that addressed the issues is by Prabheesh [24], who found a unidirectional causality runs from FPI flows to stock returns in the Indian context. In this paper, we re-examine the relationship between FPI flows and stock returns in the Indian context by incorporating the COVID-19 uncertainty index constructed by Narayan et al. [19]. The pandemic uncertainty index helps to capture the dynamic effect of COVID-19 on stock returns. In other words, the earlier papers which analyze the impact of FPI on stock returns during the COVID-19 phase assume that the inherent risk associated with the pandemic is the same across the sample period. However, the pandemic index of Narayan et al. [19] helps segregate the pandemic's relative intensity during the COVID-19 period. The contribution of the study is as follows. First, our study does the robustness check of earlier findings by analyzing the causality among stock prices and portfolio flows during the COVID-19 period. Second, we make use of the latest dataset developed by Narayan et al. [19]^1^ to examine the causality among stock markets and portfolio flows in the case of the Indian economy. From the policy implication perspective, this study will be useful for policymakers to understand the causality between stock prices and portfolio flows during the uncertainty and design a policy to improve the market sentiments and thereby attract foreign capital flows to the economy.

Section II explains the data and method, and Section III reports the empirical findings. Finally, section IV concludes.

### Data and methodology

The daily data from 31st December 2019 to 28th April 2021 is used for the empirical analysis. To proxy the stock return, we use the Nifty-50 index obtained from the website of the National Stock Exchange (NSE) index. Similarly, data for net portfolio investment flows is collected from the CEIC database. Finally, the COVID-19 uncertainty index is drawn from Narayan et al. [19]. The FPI flows are denominated in rupees (crores). To calculate the stock returns, we use the following formula as $r_{t}$=In($P_{t})-In\left(P_{t-1}\right)$*100) where *r_t_* is daily return and *P_t_* is closing prices.

In order to estimate, we deploy the Modified Wald (MWALD) Granger causality test developed by Toda and Yamamoto [34] to examine the dynamics of the COVID-19 index, FPI and stock returns. The key benefit of this approach, as compared to a simple, unrestricted vector autoregressive model (VAR), is the applicability of this method, even if the VAR is stationary or integrated of a random order. This technique makes it possible to test causality in Granger's sense by evading the unwanted outcomes related to the power and size properties of unit root and co-integration tests [37].

We estimate the following system equations to examine the causality among FPI, COVID-19 Index and stock market returns.

(6) $$ret_{t}=\propto_{1}+\sum_{j-1}^{n+d_{max}}\beta_{1j}ret_{t-j}+\sum_{j-1}^{n+d_{max}}\gamma_{1j}fpi_{t-j}+\sum_{j-1}^{n+d_{max}}\delta_{1j}cov_{t-j}+\varepsilon_{1t}$$

(7) $$fpi_{t}=\propto_{2}+\sum_{j-1}^{n+d_{max}}\beta_{2j}ret_{t-j}+\sum_{j-1}^{n+d_{max}}\gamma_{2j}fpi_{t-j}+\sum_{j-1}^{n+d_{max}}\delta_{2j}cov_{t-j}+\varepsilon_{2t}\;$$

(8) $$cov_{t}=\propto_{3}+\sum_{j-1}^{n+d_{max}}\beta_{3j}ret_{t-j}+\sum_{j-1}^{n+d_{max}}\gamma_{3j}fpi_{t-j}+\sum_{j-1}^{n+d_{max}}\delta_{3j}cov_{t-j}+\varepsilon_{3t}$$

where $ret_{t}$, $fpi_{t}$ and $cov_{t}$ represent the stock returns, foreign portfolio investment and covid-19 index at time *t. ε* is the serially uncorrelated random error terms, α denotes intercept, *n* indicates the optimum lag criteria and $d_{max}$ represent the highest order of integration. The statistical implication of $\gamma_{1j}$ and $\delta_{1j}$ in Eq. (6) indicates the causality runs from FPI to stock returns and Covid-19 to stock returns, respectively. Similarly, in Eq. (7), the statistical significance of $\beta_{2j}$, $\delta_{2j}$, show the causality from stock returns to FPI and covid-19 to FPI, respectively. Finally, the significance of $\beta_{3j}$, $\gamma_{3j}$ reveal the role of stock return and FPI on COVID-19.

### Empirical findings

Before conducting the casualty test, the stationarity conditions of the variables were tested using Narayan-Popp's [18] unit root test, and the results are presented in Table 1. The findings show that with structural breaks, all the variables are stationary at levels. Most of the breaks occurred in May 2020 (e.g., 06/05/2020), especially in the case of the Covid-19 index. The break date coincides with the decline in the uncertainty associated with the pandemic. Thus, we divide the sample period into two, i.e., from 31/12/2019 to 06/05/2020 and 07/05/2020 to 28/4/2021. Similarly, as we found all variables as stationary in levels, the maximum order of integration is set as zero ($d_{max}=0$). As a next step, we choose the optimum lag length using Akaike and Schwarz information criteria, and it found it as 6, where the VAR satisfy both stability condition and no autocorrelation issues. The Granger causality obtained from the VAR is reported in Table 2. From the table, it is clear that the null of FPI does not Granger causes stock market returns and *vice versa* is rejected during the first sample size, indicating the causality runs from both directions. This is a clear indication of the feedback trading strategy adopted by the FII during the crisis, where they sell stocks when the prices are falling and can spiral down prices.

**Table 1:** Unit Root Test Findings This table reports the unit root properties estimated by using the Narayan-Popp [18] test. Where M1 is helpful in findings two breaks at the level, and M2 is useful in identifying one endogenous break at the level and one break at the trend, respectively. The symbols *, ** and *** signify the significance at 1%, 5%, and 10% levels, respectively.

M1: two breaks in intercept					M2: one break in Intercept and one in trend			
Variables	Lag	t-stat	TB1	TB2	Lag	t-stat	TB1	TB2
**Sample (31/12/2019–28/04/2021)**								
$ret_{t}$	5	−0.544 (−3.590)	2020.05.06	2020.05.28	5	−0.824 (−5.030)**	2020.05.06	2020.05.28
$fpi_{t}$	5	−0.549 (−5.373)*	2020.04.22	2020.07.09	5	−0.799 (−6.363)*	2020.04.22	2020.07.09
$cov_{t}$	5	−0.244 (−3.530)	2020.07.30	2020.08.04	5	−0.846 (5.968)*	2020.05.06	2020.08.04
**Critical Values**								
Model			1%			5%		10%
M1 (structural breaks in intercept)			−4.731			−4.136		- 3.825
M2 (structural breaks in intercept and trend)			−5.318			−4.741		−4.430

**Table 2:** Granger Causality Test Results The table reports the Granger causality findings. The findings are based on *m* = 6 and *d_*max= 0. The $H_{0}$ hypothesis indicates that there is no causal relationship and the $H_{1}$ hypothesis indicates that there is a causal relationship. The asterisks *,**, and *** denotes significance level at 1%, 5% and 10%, respectively.

Causality Design	T-Stats	P-values
**COVID-19 sample 1 (31/01/2019–06/5/2020)**		
FPI to Stock Return	14.705	0.022**
Stock Return to FPI	11.824	0.066***
COVID-19 to FPI	12.243	0.056***
FPI to COVID-19	0.939	0.987
COVID-19 to Stock Return	6.021	0.420
Stock Return to COVID-19	6.024	0.450
**COVID-19 sample 2 (07/5/2020–28/04/2021)**		
FPI to Stock Return	8.032	0.530
Stock Return to FPI	51.841	0.000*
COVID-19 to FPI	10.068	0.345
FPI to COVID-19	13.549	0.139
COVID-19 to Stock Return	12.044	0.210
Stock Return to COVID-19	7.097	0.627

Similarly, the null hypothesis Covid-19 does not cause FPI is rejected, indicating Covid-19 induced uncertainty significantly predicts the FPI flows. However, the causality from FPI to Covid-19 is not statistically significant. Similarly, the findings also suggest that the Covid-19 index does not predict the stock return significantly. Overall findings during this period indicate that the impact of Covid-19 on stock return is strong through the FPI, supporting the significant withdrawal of capital from the Indian stock market by the FPI during the early days of the pandemic.

However, during the second period, from 07/05/2020 to 28/4/2021, all null hypotheses are not rejected except ‘Stock returns Granger causes FPI’. This is clearly indicating that during this period, the stock market revival attracted foreign investors to the Indian economy. Further, these findings also indicate the supporting policies of the Govt. of India, both fiscal and monetary policies, helped improve the market sentiments and attract foreign investments.

## Conclusions

This study re-examined the causal association between FPI inflows and stock market returns using the COVID-19 uncertainty index proposed by Narayan et al. [19] for India. Our findings show that in the course of the pandemic, the stock market dynamics with FPI are determined by the degree of pandemic uncertainty. Furthermore, we found that during the early period of the pandemic, from Dec 2019 to May 2020, there was a bi-directional causality run from stock returns to FPI, supporting the substantiation of the feedback trading strategy adopted by the overseas portfolio investors. However, during the later period, FPI flows are primarily driven by stock returns, indicating the govt. policy measures help to improve the market sentiments and thereby attract foreign capital flows to the economy.

## Funding source declarations

The authors declare that no funding has been received to carry out this research work.

## Declaration of Competing Interests

This article is submitted to MethodsX as a special issue in COVID Research Robustness. The authors declare that they have no known competing financial interests or personal relationships that could have appeared to influence the work reported in this paper.

## Data Availability

Data will be made available on request.

## Footnotes

1 The uncertainty index constructed by Narayan et al. [19] captures the uncertainty associated to the pandemic by utilizing the 45 most popular newspapers worldwide and using the 327 keywords related to a different aspect of COVID-19. Further, this uncertainty index is specially designed for COVID related uncertainty, hence it is sophisticated than other existing uncertainty index used in the literature such as volatility index.
